# P02.97. Lipoic acid supplementation induces a transient stress response and improves episodic memory and cholesterol efflux in humans

**DOI:** 10.1186/1472-6882-12-S1-P153

**Published:** 2012-06-12

**Authors:** D Keith, J Butler, B Bemer, J Mogle, J Monette, J Hair, J Heinecke, M Sliwinski, T Hagen

**Affiliations:** 1 Linus Pauling Institute, Oregon State University, Corvallis, USA; 2 Pennsylvania State University, State College, USA; 3 University of Washington, Seattle, USA

## Purpose

Lipoic acid (LA) shows promise as a beneficial micronutrient in improving health, particularly in the elderly. Clinical and *in vitro* reports show that LA induces endogenous antioxidants and acts as an anti-inflammatory agent. LA also increases nerve conductance, improves diabetes-induced polyneuropathies, and remediates the age-associated cognitive decline in canines. Furthermore, LA significantly improves hypertriglyceridemia and glucose handling. From our pre-clinical research we have found that LA primarily influences three areas of health: cognition, stress response, and lipids. This study examines the effects of LA on human subjects in components of each of the three aforementioned areas of health.

## Methods

This study utilized acute treatments with LA supplements as well as chronic supplementation in an 8 week, double-blind placebo-controlled cross-over trial in human volunteers. The subjects were grouped into young (18-45 years) or elderly (~79 years), and all subjects were administered the R-enantiomer of LA in the form of oral supplements. This was a small pilot study with 2-6 subjects in each experiment.

## Results

Results from the backward letter span and paired associates cognitive tests indicate that LA improved verbal episodic memory in the elderly, but did not improve short-term memory in the young or elderly. Chronic LA altered stress response systems as indicated by a transient increase in salivary cortisol and aldehyde dehydrogenase 3A1. Chronic LA also increased the amount of cholesterol taken up by high-density lipoproteins, particularly in the elderly subjects.

## Conclusion

LA may be useful as a complementary nutraceutical agent to improve cholesterol efflux and select memory processes, particularly in the elderly. Continuous supplementation with LA induces a transient catabolic state, as well as a transient stress response. Thus, LA may function as a hormetic agent by inducing an initial stress that primes the system to efficiently respond to future toxicological insult.

